# Morphostructural, Phaneroptic Characterization, and Zoometric Indices of Creole Cattle (*Bos taurus*) from the High Andean Region of Ayacucho, Peru

**DOI:** 10.3390/ani16132101

**Published:** 2026-07-07

**Authors:** Mijail Contreras Huamaní, César Jorge Mendoza Leiva, Jhoel Kevin Alvaro Peralta, Hamilton Guzman Santaria, Walter Palomino-Guerrera, Hurley Abel Quispe-Ccasa

**Affiliations:** 1 Estación Experimental Agraria Canaán, PROGAN Project, Instituto Nacional de Innovación Agraria, Ayacucho 05002, Peru; cesar.jorge.mendoza.leiva@gmail.com (C.J.M.L.); hmltnguzman@gmail.com (H.G.S.); mvpalomino92@gmail.com (W.P.-G.); 2 Escuela Profesional de Medicina Veterinaria, Universidad Nacional de San Cristóbal de Huamanga, Ayacucho 05000, Peru; jhoel.alvaro.24@unsch.edu.pe; 3 Dirección de Investigación y Desarrollo Tecnológico, PROGAN Project, Instituto Nacional de Innovación, Lima 15024, Peru; hurleyabelqc@gmail.com

**Keywords:** cattle, morphometry, phenotypic variation, adaptation, high altitude

## Abstract

Morphostructural characterization of Creole cattle is essential for assessing the diversity and productive potential in the highland livestock systems. The study was conducted in Chuschi and Chipao districts, at 3800 m above sea level, with the objective of morphostructurally characterizing 154 Creole cows. Seventeen phaneroptic variables and twenty-one morphometric variables were recorded. The multivariate analysis identified three cattle biotypes. Based on proportionality, compactness, and cannon bone load indices, Biotype 1 (16.9%) comprised more compact, robust, and heavier animals with a greater tendency toward meat production aptitude, followed by Biotype 2 (48.1%) and Biotype 3 (35.1%), which were more slender but with a stronger bone structure. No association was found between biotypes and phaneroptic characteristics, except for teat type (*p* < 0.05). The proportions of red, black, dull black, qosca, and roan coat colors were common across all biotypes; however, Biotype 1 predominated in callejón (15.38%), Biotype 2 in qosne (8.11%) and in other less frequent coat colors (10.81%), while Biotype 3 predominated in dark roan (16.67%) and jet black (11.11%). In the high-Andean region of Ayacucho, three subpopulations of Creole cattle were identified with potential for beef production, dual-purpose use, and adaptation to rugged terrain, highlighting the need to investigate their genetic diversity.

## Simple Summary

High-Andean Creole cattle have become an endemic resource of great socioeconomic interest in remote regions due to their adaptability and phenotypic–genotypic diversity, which should be conserved and sustainably utilized. The objective of this study was to carry out a morphostructural characterization of Creole cows in the districts of Chuschi and Chipao, Ayacucho, Peru, at 3800 m above sea level, through the recording of body measurements and qualitative traits. Three Creole cattle biotypes were identified that were not associated with the district and specific qualitative traits. Biotype 1 consisted of more compact and heavier cows with a greater tendency toward beef production aptitude, followed by Biotypes 2 and 3, which were slimmer cows but with a stronger skeletal structure. In addition, a higher percentage predominance of the callejón coat color was observed in Biotype 1, qosne and the group of less frequent coat colors in Biotype 2 and dark roan and jet black in Biotype 3.

## 1. Introduction

Cattle were introduced to the American continent during the Spanish colonization beginning in the 15th century, and since then they have adapted over many generations with relatively efficient productive performance, higher fertility rates, greater longevity, and tolerance to diseases and harsh conditions. This adaptation has occurred across a wide range of geographic environments characterized by low-quality pastures and extreme temperature and humidity conditions [1,2]. Creole cattle are therefore considered a zoogenetic resource of broad diversity.

Creole cattle are of great socioeconomic and cultural importance for rural subsistence-based communities, as they are versatile in the production of milk, meat, and draft power in steep terrains where agricultural machinery cannot operate. Despite exhibiting modest productive levels, they show good potential in intensive fattening systems and represent an alternative for concentrating genes to develop a local biotype with high productive capacity, while contributing genetic traits such as hardiness, maternal ability, adaptation to high altitude [3], and high digestive efficiency [4].

However, Creole cattle are increasingly experiencing genetic erosion due to the introduction of highly productive exotic breeds with high management requirements and low adaptability to harsh environments [5], often through crossbreeding implemented without specific genetic improvement plans [1]. Many South American countries have opted to introduce specialized breeds with the expectation of improving meat or milk production efficiency; nevertheless, this trend may lead to the loss of the genetic richness of locally adapted cattle, potentially reaching the point of genotype extinction, as reported in El Salvador [6]. Furthermore, there is limited information regarding the zoogenetic valuation of Creole cattle and their phenotypic characteristics and productivity level. However, field experience suggests that production is minimal due to their adaptation to challenging environmental conditions, but that this adaptation represents an advantage for this animal byotipe [7], contributing to its survival.

It is therefore essential to consider the appropriate use of specialized breed genotypes in Creole cattle in order to ensure their sustainable utilization. Phenotypic characterization based on physical and morphological description constitutes one of the initial stages for effective management of zoogenetic resources [8]. Morphometric characterization of Creole cattle is useful for assessing their diversity and productive potential. At the regional level, several studies have reported the characterization of Creole cattle, describing a wide diversity of traits [9,10,11,12,13]. Others report traits shaped by selection and crossbreeding [14,15,16,17,18,19,20]. Furthermore, a comprehensive morphostructural characterization must also consider the harmony and uniformity of the population. Harmony reflects the proportional relationship among body regions in accordance with the productive aptitude of the animal, while uniformity indicates the degree of phenotypic homogeneity that defines a breed or biotype [21]. Both criteria are essential for objectively interpreting morphostructural data and determining the degree of adaptive consolidation of a local population such as Creole cattle.

Under the high Andean conditions of Peru, Creole cattle have been naturally selected for their adaptability and hardiness; however, their phenotypic variability and morphostructural characteristics have not yet been fully described. The objective of the present study was to perform a morphostructural characterization, based on body measurements and zoometric indices, of Creole cows of reproductive age in the high Andean region of Peru.

## 2. Materials and Methods

### 2.1. Ethical Statement

No activities that could cause unnecessary stress to the animals were carried out in this study; therefore, approval from an institutional ethics committee was not required. Nevertheless, the study adhered to the recommendations of the Peruvian Animal Protection and Welfare Law (Law No. 30407) and to the guidelines of the ARRIVE 2.0 Ethical Code for animal experiments, available at http://www.nc3rs.org.uk/ARRIVEchecklist (accessed on 15 January 2026).

### 2.2. Study Area

**Figure 1:**
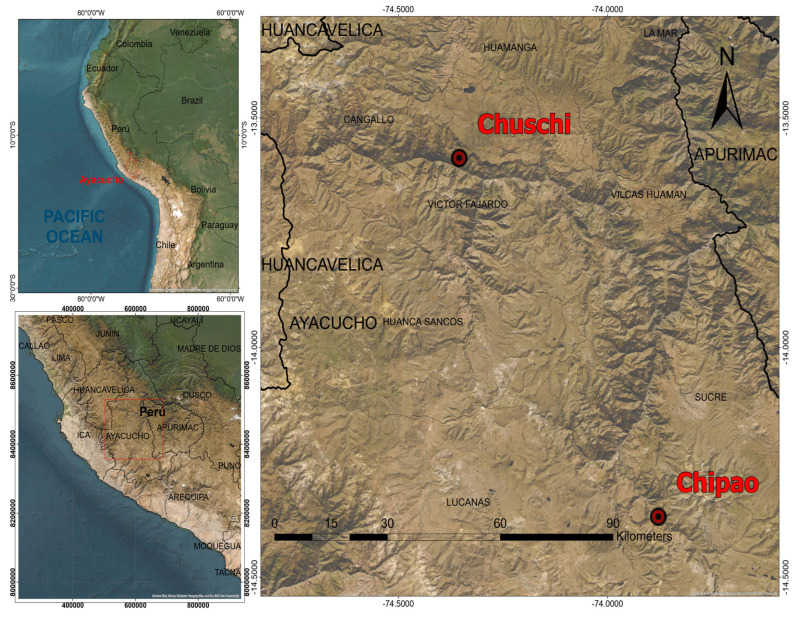
Location of the study area in the Department of Ayacucho. Chipao District in the Lucanas Province, and Chuschi District in the Cangallo Province.

The study was conducted in the districts of Chuschi and Chipao, geographically located between 2680 and 5500 m above sea level in the Ayacucho Department in Peru (Figure 1). The districts of Chuschi and Chipao concentrate cattle populations of 10,052 and 12,000 heads, respectively. The regional climate is mainly divided into two seasons: the rainy season (December to March) and the dry season (May to September). The area records a mean annual temperature of 9.96 °C and a relative humidity of 73.4%.

### 2.3. Breading System

In this study, animals were considered Creole cattle based on phenotypic, historical, and management criteria for Andean Creole cattle, according to Rojas-Espinoza et al. [13]. Specifically, animals were identified by their long-term adaptation to high-altitude Andean conditions, their maintenance under extensive production systems with natural pasture feeding, the absence of controlled crossbreeding with specialized foreign breeds, and the presence of characteristic phenotypic traits, including diverse coat color patterns and body conformation typical of Creole populations.

Farmers wean calves at six months of age and do not apply any selection criteria for mating, which occurs mainly through natural service. Regular vaccination programs are not implemented, except for immunization against blackleg and occasional internal deworming campaigns. Cattle are mainly used for meat production, milk production (ranging from 450 to 1500 L per lactation), and draft power.

### 2.4. Population, Sample and Design

The Ayacucho Department has a total of 430,462 heads of cattle [22]; however, the specific population size of Creole cattle in the study areas, with characteristics described in the previous paragraph, is unknown. The sample size was determined using the formula for an unknown population [23]:

$$n=\frac{\left(Z^{2}*p*q\right)}{e^{2}}$$

where *n* = sample size, *Z* = 95% confidence level (1.96), *p* = probability of occurrence of the event (50%), *q* = probability of non-occurrence of the event (50%), and *e* = margin of error (8%). The resulting sample size was 154 Creole cattle.

The study followed a descriptive–explanatory cross-sectional design. Samples were distributed between the districts of Chuschi (n = 95 females) and Chipao (n = 59 females) and were randomly selected, ensuring that all cattle in the total population had the same probability of being included in the sample. Because breeders in the region do not keep records, animal age was determined by dentition, including adult females with more than four permanent teeth (over three years of age), while females younger than two years with positive pregnancy status were excluded.

### 2.5. Phaneroptic Traits

**Figure 2:**
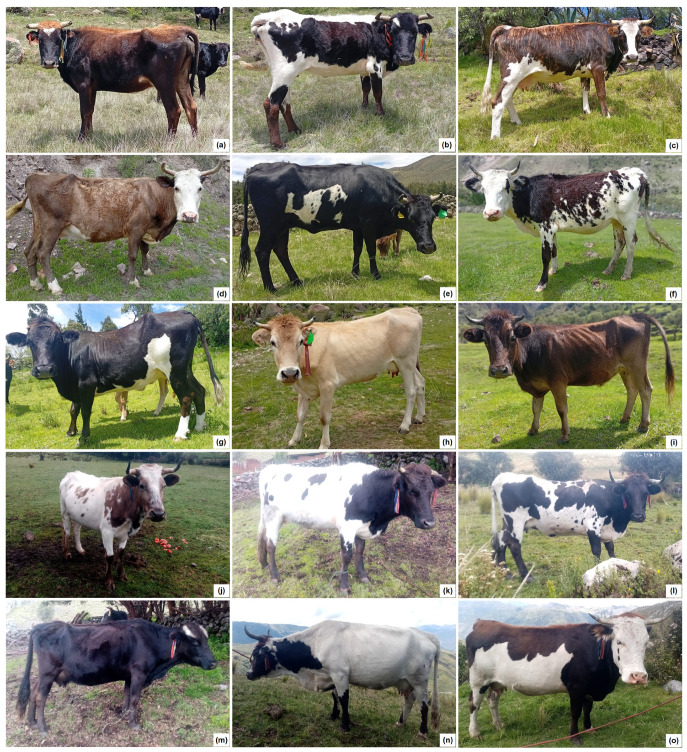
Coat colors of Creole cattle from the high Andean region of Ayacucho: Qosca frontino (**a**), Black callejón lucero (**b**), Roman frontino (**c**), Qosne mascorona (**d**), Black bragado (**e**), Roan mascorona (**f**), Black bragado (**g**), Bayo (**h**), Red (**i**), Qosne omara (**j**), White omara (**k**), Mora callejón (**l**), black (**m**), White omara (**n**), Qosca Callejona mascarona (**o**).

Seventeen phaneroptic characteristics were recorded, including coat color variability (Table 1, Figure 2).

### 2.6. Zoometric Variables

Twenty-one zoometric variables were recorded (Table 2). Live weight was estimated using a bovine weight tape 0405, while zoometric measurements were recorded in centimeters (cm) using a measuring tape (Anvil, model 96405; Beijing, China) and a zoometric measuring stick. The latter followed an ad hoc design consisting of a 155 cm × 10 cm × 1 cm board fixed perpendicularly to the end of a second board measuring 30 cm × 5 cm × 2 cm, forming an “L” shape, and a third board of 30 cm × 5 cm × 2 cm that slid along the scale of measurements on the second board. Animals were firmly restrained on a flat, level surface, standing squarely on all four limbs with the head lowered, so that the poll and the withers were at the same height.

### 2.7. Zoometric Indices

From the zoometric measurements obtained, nine zoometric indices were calculated [26] mendez. Ethnological indices were calculated to describe body proportions and general conformation (Body Index, Pelvic Index, and Proportionality Index), productive indices are associated with productive aptitude, particularly meat or dual-purpose potential (Compactness Index, and Dactylo-Thoracic Index), and functional indices that provide information related to skeletal strength, locomotion, and the animal’s capacity to support body weight under specific environmental conditions (Transversal Pelvic Index, Longitudinal Pelvic Index, Relative Cannon Thickness Index, and Cannon Load Index) (Table 3). These indices were analyzed in order to identify the productive orientation of the animal biotypes.

### 2.8. Statistical Analysis

The database was organized and initially analyzed using descriptive statistics. Zoometric variables were subjected to descriptive analysis to determine the mean and standard error (SE) according to district of origin. The coefficient of variation (CV) was calculated to assess the uniformity of the animals within the population. Data were tested for normality by the Kolmogorov–Smirnov test and homoscedasticity by the Levene test. Differences according to district of origin were analyzed using ANOVA, and mean comparisons were performed using the Duncan test. Data that did not meet normality and homoscedasticity assumptions were analyzed using the nonparametric Kruskal–Wallis test, and mean comparisons were performed using the Mann–Whitney U test. These analyses were carried out using the IBM SPSS v.15.0 program.

Spearman correlation coefficients were calculated between fifteen zoometric variables of the morphostructure and nine indices separately, to evaluate the level of harmony of the animals within the population by means of the proportion of positive and significant correlations (*p* < 0.05). A principal component analysis (PCA) was conducted to reduce the dimensionality of the 15 zoometric variables. The Kaiser–Meyer–Olkin (KMO) test value (0.85) and Bartlett’s test of sphericity (*p* < 0.001) were used to verify the suitability and reliability of the factor analysis. Subsequently, a K-means cluster analysis was performed to group animals according to their zoometric measurements, and clusters were validated using permutational analysis of variance (*p* < 0.001).

Finally, descriptive statistics of zoometric variables between identified biotypes were analyzed using ANOVA with Duncan’s test or the Kruskall–Wallis and Mann–Whitney U tests with Bonferroni correction, according to the goodness of fit of the variables. Associations between biotypes and district of origin and body condition were evaluated using the χ^2^ test to rule out clustering bias based on these variables. Phaneroptic variables were analyzed using contingency tables and also evaluated using the χ^2^ test. All multivariate analyses were performed in the R v.4.4.3 statistical software.

## 3. Results

### 3.1. Zoometric Characterization

Twenty-one zoometric variables were analyzed in 154 Creole cows from the districts of Chuschi and Chipao in the Ayacucho Department (Table 4). Animals exhibited high variability in Horn Length (26.32%), Neck Length (13.0%), Withers Width (13.92%), Chest Width (10.36%), Udder Depth (18.23%), Teat Length (26.94%), Teat Diameter (22.57%), Thigh Width (20.32%), and Live Weight (21.41%), pero eran más uniformes en el resto de variables (<10%). The zoometric measurements Neck Length, Neck Circumference, Withers Height, Withers Width, Thoracic Perimeter, Teat Length, Front Cannon Bone Circumference, Rear Cannon Bone Circumference, and Live weight, showed significant differences between districts (*p* < 0.05). The district of Chuschi concentrates heavier Creole cattle with good thoracic development and well-developed fore and hind cannons that provide adequate skeletal support. In contrast, cattle from Chipao showed greater stature, body length, and a larger rump area, but lower live weight.

**Figure 3:**
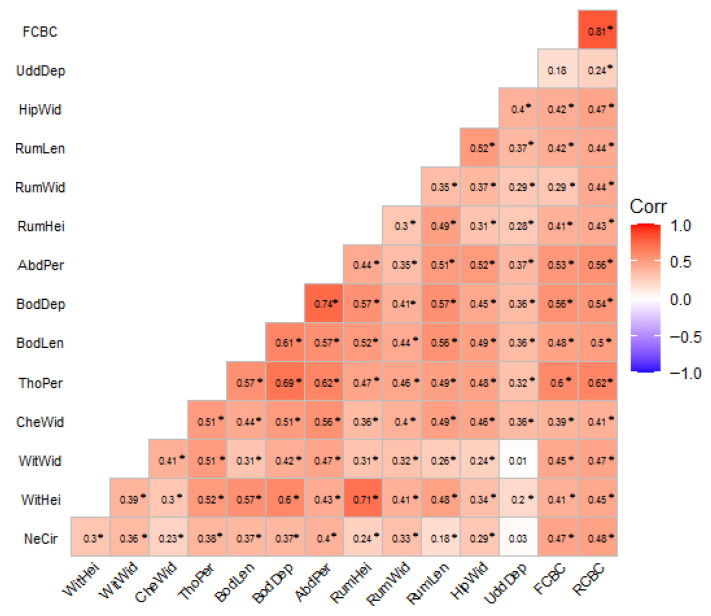
Correlation among zoometric variables of Creole cows using Spearman correlation coefficients. Neck Circumference (NeCir), Withers Height (WitHei), Withers Width (WitWid), Chest Width (CheWid), Thoracic Perimeter (ThoPer), Body Length (BodLen), Body Depth (BodDep), Abdominal Perimeter (AbdPer), Rump Height (RumHei), Rump Width (RumWid), Rump Length (RumLen), Hip Width (HipWid), Udder Depth (UddDep), Front Cannon Bone Circumference (FCBC), Rear Cannon Bone Circumference (RCBC). (*) The correlation is significant at the *p* < 0.05 level.

In Figure 3, the correlation coefficients ranged from 0.01 to 0.81, representing positive relationships. The 15 morphostructural variables generated 105 correlations, of which 97.14% (103/105) were positive and statistically significant (*p* < 0.05), suggesting a high degree of morphostructural harmony in the studied population. The highest correlation was observed between FCBC with RCBC (r = 0.81) and Body Depth (BodDep) with Abdominal Perimeter (AbdPer) (r = 0.74). The zoometric measurements, Withers Height (WitHei), Withers Width (WitWid), Chest Width (CheWid), Thoracic Perimeter (ThoPer), Body Length (BodLen), Body Depth (BodDep), Abdominal Perimeter (AbdPer), Rump Height (RumHei), Rump Width (RumWid), Rump Length (RumLen), Hip Width (HipWid), FCBC, and RCBC showed strong correlations among themselves. These variables exhibited moderate correlations with Udder Depth (UddDep) and Neck Circumference (NeCir).

**Figure 4:**
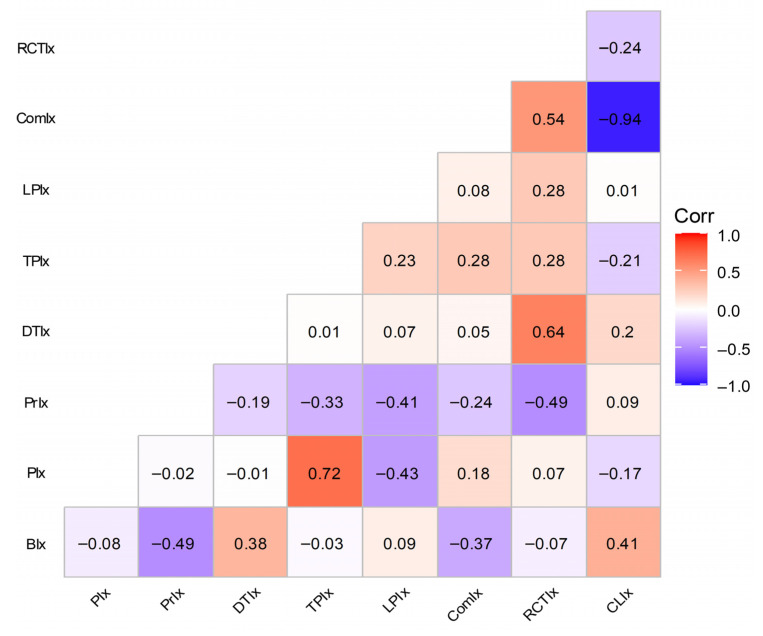
Correlation among zoometric indices of Creole cows using Spearman correlation coefficients. Body Index (Bix), Pelvic Index (Pix), Proportionality Index (PrIx), Dactylo-Thoracic Index (DTIx), Transversal Pelvic Index (TPIx), Longitudinal Pelvic Index (LPIx), Compactness Index (ComIx), Relative Cannon Thickness Index (RCTIx), Cannon Load Index (CLIx).

Regarding the correlations among zoometric indices, a strong positive correlation was found between the Pelvic Index (PIx) and the Transversal Pelvic Index (TPIx) (r = 0.72), while a strong negative correlation was observed between the Compactness Index (ComIx) and the Cannon Load Index (CLIx) (r = −0.94) (Figure 4).

### 3.2. Principal Components Analysis and Clustering

**Figure 5:**
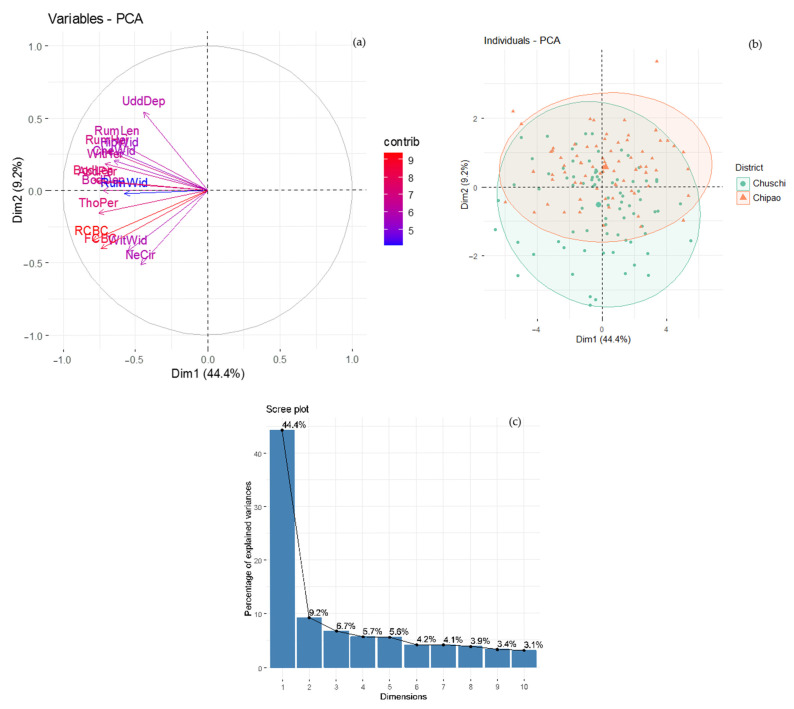
Contribution of 15 zoometric variables to the variability of Creole cows (**a**), Projection of Creole cows in multivariate space (**b**), and Cumulative explained variance of zoometric measurements of Creole cows obtained by PCA (**c**).

A principal component analysis (PCA) was performed to reduce the dimensionality of 15 zoometric variables, excluding Horn Length, Neck Length, Teat Length, Teat Diameter, and Thigh Width due to their lower contribution and relevance to data variability. The adequacy of the data for factor analysis was confirmed by the Kaiser–Meyer–Olkin (KMO) test (0.85) and Bartlett’s test of sphericity (*p* < 0.001). Fifteen dimensions were identified, of which the first five explained 71.6% of the cumulative variance (Figure 5a–c).

Accordingly, the rotated component matrix of the first five dimensions was extracted to determine the contribution of each zoometric variable to each dimension (Table 5). Thoracic Perimeter, Body Length, and RCBC defined Dimension 1, representing variables related to overall robustness; Neck Circumference, Rump Length, and FCBC defined Dimension 2, representing neck and pelvic robustness; Withers Height, Rump Height, and Udder Depth defined Dimension 3, representing general body size; Rump Width and Hip Width defined Dimension 4, representing pelvic development; and Withers Width, Chest Width, Body Depth, and Abdominal Perimeter defined Dimension 5, representing body depth and width.

**Figure 6:**
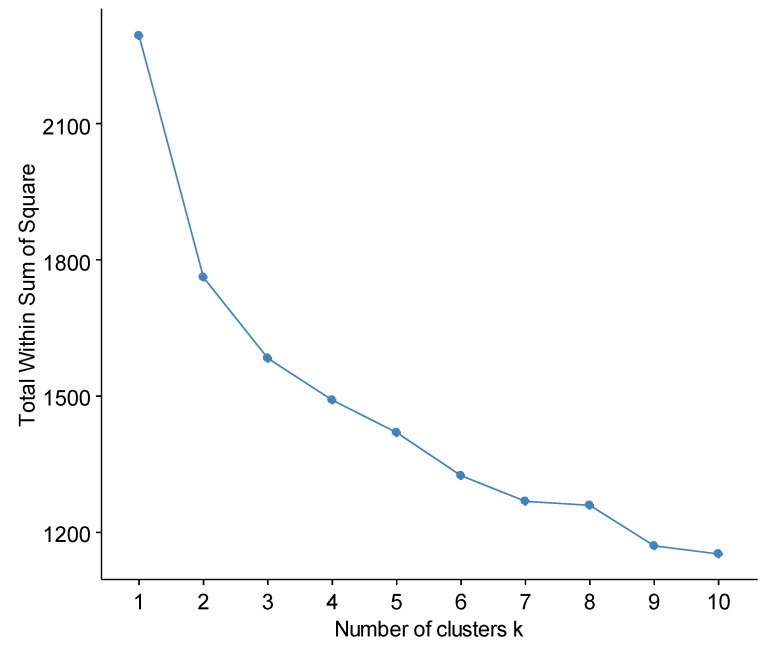
Elbow plot for determining the optimal number of clusters.

**Figure 7:**
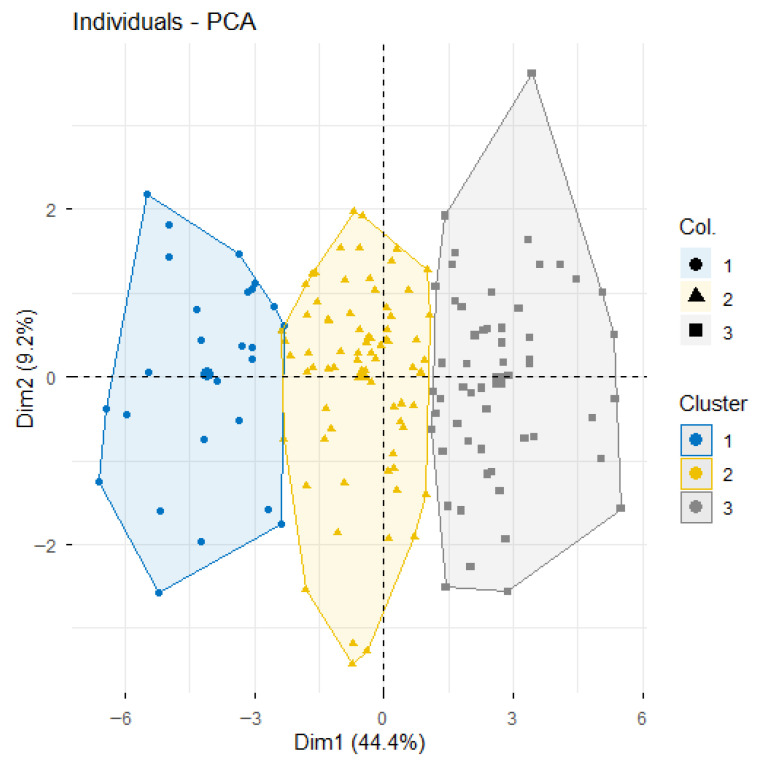
Clusters formed by the k-means method in Creole cows from the high Andean region of Ayacucho Department.

To classify individuals, the optimal number of clusters was determined using the elbow method (Figure 6), and classification into three clusters was performed using the k-means method (Figure 7). A permutational multivariate analysis of variance (PERMANOVA) was conducted to evaluate the effectiveness of the k-means clustering in classifying individuals based on their zoometric traits, yielding a *p*-value < 0.001 with 999 permutations (Table 6).

According to Table 7, of the 154 individuals evaluated, 26 cows formed Cluster or Biotype 1 (16.9%), 74 cows formed Biotype 2 (48.1%), and 54 cows formed Biotype 3 (35.1%). Mean values of zoometric variables for each biotype are also presented. All variables showed significant differences among biotypes (*p* < 0.05 and *p* < 0.01), except for Horn Length, Neck Length, and Teat Diameter (*p* > 0.05). Cows classified as Biotype 1 were heavier and longer animals with well-developed thoracic and pelvic regions and greater stature, whereas Biotype 2 showed similarities in neck and pelvic robustness. Biotype 3 consisted of lighter cows with smaller body proportions compared to the other two biotypes (*p* < 0.05 and *p* < 0.01).

Based on the zoometric variables, zoometric indices were calculated for the three identified biotypes (Table 8), of which only ComIx and CLIx showed significant differences among groups (*p* < 0.05). According to the Body Index (BIx), cows of Biotypes 2 and 3 were more brevilinear (<85) than those of Biotype 1; meanwhile, Biotypes 1 and 3 exhibited a pelvis wider than long compared to Biotype 2. The Dactylo-Thoracic Index (DTIx) suggested greater meat production aptitude in Biotype 1 compared to Biotypes 2 and 3. The Transversal Pelvic Index (TPIx) and Longitudinal Pelvic Index (LPIx) indicated greater meat aptitude in Biotypes 1 and 2. The Relative Cannon Thickness Index (RCTIx) indicated greater limb robustness in Biotype 1 compared to Biotypes 2 and 3, although these differences were not significant (*p* > 0.05). According to the Compactness Index (ComIx) and the Cannon Load Index (CLIx), Biotype 1 comprised more compact, heavier, and more robust animals, but with a relatively lighter bone structure and a lower threshold for supporting high specific weights. In contrast, Biotype 3 consisted of more slender animals with lower relative body weight but stronger bones to support their body mass (*p* < 0.01). According to Table 8, of the 154 individuals evaluated, 26 cows formed Cluster or Biotype 1 (16.9%), 74 cows formed Biotype 2 (48.1%), and 54 cows formed Biotype 3 (35.1%). All variables showed significant differences among biotypes (*p* < 0.05 and *p* < 0.01), except for Horn Length, Neck Length, and Teat Diameter (*p* > 0.05).

No significant association was found between the identified biotypes and categorical variables such as district of origin, body condition score, and number of calvings per cow, suggesting that biotype classification is independent of these categorical factors (Table 9).

### 3.3. Phaneroptic Characteristics According to Biotypes

The association between biotypes and the recorded phaneroptic variables of Creole cows was analyzed. Most variables were not significantly associated with the identified biotypes (*p* > 0.05), except for teat type (*p* < 0.05). Cylindrical teats predominated in Biotype 1 (53.8%) and 2 (53.1%), whereas funnel-shaped teats were more frequent in Biotype 3 (40.7%) (Table 10). Overall, 30.5% of Creole cattle exhibited a solid coat pattern, 62.3% were bicolor, and 7.1% were tricolor. Animals with a straight frontonasal profile, short-horn type, dark mucosa, and light-colored udders and teats predominated.

Coat color frequencies were determined according to the identified biotypes, and the association was significant (*p* = 0.009). Similar proportions of Red, Black, Dull Black, Qosca, and Roan coat colors were observed across the three biotypes (Table 11). However, Biotype 1 showed predominance of the Callejón coat color (15.38%), whereas Biotype 2 showed predominance of the Qosne coat color (8.11%) and other less frequent coat colors (10.81%), including Bayo, Pillco, Qallawa, Puca moro, Umara, and Tres Pelos. In Biotype 3, in addition to the common coat colors, Dark roan (16.67%) and Jet Black (11.11%) predominated.

## 4. Discussion

### 4.1. Zoometric Characteristics

Phenotypic characterization based on morphological and phaneroptic descriptions constitutes the first step toward implementing conservation programs and the sustainable use of animal genetic resources to develop genotypes and/or breeds specialized in milk production, meat production, or dual-purpose sustainable systems [8,13]. In this study, the average live weight of Creole cattle was 368.76 ± 6.45 kg, which is slightly lower than that reported for Creole cattle from the Puno region [13] and Amazonas [12], but higher than values reported for Creole cattle from Ancash [10]. These lower weights compared with other latitudes may be attributed to differences in environmental conditions, management systems, or feeding practices. In the high-Andean region, Creole cattle generally graze on pastures with low nutritional value. In comparable studies, cows from the present study were heavier than Creole cattle from Cusco [30], Creole cattle from Ecuador [31], and Mexican Mixteco Creole bulls [20], and showed similar weights to Sanmartinero Creole cattle [32]. However, they were lighter than Blanco Ojinegro Creole cattle [17].

Creole cattle from Puno [13] and Amazonas [12] were slightly larger and longer than those evaluated in this study. Likewise, Creole cattle from Santa Elena Province in Ecuador exhibited greater height at withers and a finer conformation [7]; similarly, Patagonian Creole cattle [33] and Blanco Ojinegro Creole cattle [17] were reported to have greater thoracic girth and body length. In contrast, Creole cattle from Loja Province in Ecuador [31] and Ethiopian indigenous cattle [27] showed smaller body dimensions.

The rump surface of the Creole cattle evaluated in this study was narrower (39.49 ± 0.26 cm) and shorter (44.74 ± 0.27 cm) than that reported for cattle from Puno [13] and Amazonas [12] in Peru, as well as for Creole cattle from Santa Elena Province in Ecuador [7]. These differences may be attributed to variations in management and feeding practices, as well as to selection and breeding processes, since these traits show moderate to high heritability [3]. Conversely, Creole cattle from Ancash [10] and Loja Province in Ecuador [31] exhibited similar proportions to those observed in the present study. Meanwhile, Creole cattle from Mexico [20] and indigenous cattle from Ethiopia [27] and Indonesia [28] showed slimmer and shorter rumps. Although studies on indigenous cattle from Ethiopia [27] and Indonesia [28] provide useful references regarding morphostructural variability under different environmental conditions, these populations are not classified as Creole cattle. The term “Creole cattle” is exclusively used for populations derived from Iberian cattle introduced into the Americas. Therefore, these comparisons should be interpreted with caution. These morphostructural differences among American [20,31], Asian and African populations [26,27] are related to non-standardized genetic manipulation by farmers and adaptive strategies under variable agroecological, functional, nutritional, and genetic conditions [34,35,36,37].

### 4.2. PCA and Cluster Analysis

Creole cattle are present in most South American countries, where they have evolved and adapted to diverse agroecological environments such as mountainous and Patagonian steppes, and tropical and subtropical forests [38]. Several studies report high genetic diversity and phenotypic variability in Creole cattle [13,31,39]; however, studies focused on population characterization and identification of morphostructural aptitudes as a basis for conservation remain scarce. In the present study, three Creole cattle biotypes were identified in the high-Andean region. Biotype 1 was characterized by greater body weight and size, followed by Biotypes 2 and 3, which were lighter and smaller. Similar classifications have been reported in Amazonas [12], Puno [13], and Ecuador [31], where three and four biotypes were identified, respectively, including coat color and hair characteristics.

Creole cattle from the high-Andean region can be classified as a medium-sized biotype, similar to those from Puno and Amazonas, although these populations exhibit greater body length. Only Creole cattle from Santa Elena Province, Ecuador [7], may be considered large-sized biotypes. Whereas Creole cattle from Ancash [10], Ecuador [31], Mexico [20], Ethiopia [27], and Indonesia [28] can be classified as small- to medium-sized biotypes, similar to those observed in this study. This wide zoometric variability may be associated with genetic selection [40], adaptation to environmental and nutritional conditions [34], and the use of external genetic material without structured breeding programs.

Based on the body index, animals can be classified as brevilineal (<85), mesolineal (86–89), or longilineal (>90), with lower values indicating more compact animals [20]. In this study, the animals were classified as brevilineal with a meat-oriented conformation. Brevilineal animals are characterized by greater longitudinal dimensions with respect to height, resulting in a rectangular body shape [21]. Similar classifications have been reported for Puno Creole cattle [13], Cusco Creole cattle [30], Santa Elena Peninsula Creole of Ecuador [7], Mexican Mixteco Creole [20], Venezuelan Limonero Creole [2], Colombian Blanco Ojinegro Creole [17], Sanmartinero Creole [32], and the Guatemalan Barroso Salmeco Creole [41]. In contrast, Creole cattle from Ancash, Peru [10] and Creole cattle of Southern Ecuador [28] were considered dual-purpose biotypes as mesosolineal classification, while Patagonian and Northeastern Argentine Creole cattle were regarded as dairy-oriented due to their longilineal body index [33].

Higher pelvic and dactylo-thoracic index values indicate meat-oriented biotypes with good maternal aptitude, and values above 33 are associated with a tendency toward beef production [20,21]. The Creole cattle in this study showed slightly lower indices than those reported for the Puno, Cusco, and Ancash Creole cattle [10,13,30], Venezuelan Limonero Creole [2], Blanco Ojinegro Creole [17], and the Argentine Patagonian Creole [33], but showed similar values to the Mexican Mixteco [20] and Guatemalan Barroso Salmeco Creole cattle [41]. Overall, these cattle can be considered brachypelvic, with a pelvis longer than wide, which is associated with greater muscle mass and ease of calving [17,21,31].

Lower proportionality index values indicate a compact, rectangular biotype [31,42]. Creole cattle in this study exhibited higher values than those reported for Puno and Ancash. Accordingly, cattle from this region may be classified as a dual-purpose biotype with a tendency toward meat production [35]. However, low dactylo-thoracic index values (<11) indicate a fine skeletal structure, characteristic of dairy-type animals [42]. High relative cannon thickness index values observed in this study suggest robust limbs, typical of beef cattle, with good balance and adaptation to long-distance walking under grazing conditions [31,43,44]. High values for the transverse pelvic index (>33) and longitudinal pelvic index (>37) are associated with a stronger inclination toward meat rather than dairy aptitude [20]. In this study, all identified biotypes may possess a greater meat aptitude, as they exceeded these established thresholds.

Overall, Creole cattle from this region are brevilineal, compact, well-balanced, with a wide and long rump and long limbs that facilitate grazing mobility, corresponding to a dual-purpose biotype with a marked tendency toward meat production. This biotype likely results from several decades of adaptation to diverse altitudinal conditions, including mountainous steppes, inter-Andean valleys, and high-altitude zones, which conditioned the shaping of this structure in order to improve its survival.

### 4.3. Phaneroptic Traits

Qualitative traits have limited direct influence on production; however, they represent important phenotypic characteristics reflecting centuries of adaptation [45]. Creole cattle are characterized by distinctive phaneroptic traits, including coat color variability, horn types [46], pigmentation, udder and teat types, among others. In this study, composite coat colors predominated, followed by solid colors. In contrast, a similar study in Ecuador reported predominance of all three coat color categories (solid, composite, and mixed) [31]. Bayssa et al. [47] indicate that coat color is associated with climatic adaptation; light-colored cattle exhibit greater heat tolerance and improved productive performance [48,49], whereas dark-coated cattle are more resistant to cold due to greater solar energy absorption [50].

Qualitative traits such as horn and ear characteristics play roles in thermoregulation and defense [51]. Creole cattle in this study were characterized by a straight frontonasal profile, short horns, dark-colored mucosae, and dark hooves. Most animals had rounded, light-colored udders with cylindrical, light-colored teats, although Riera-Nieves et al. [52] recommend funnel-shaped teats as indicators of high milk production. These adaptive traits are related to natural selection, agroecological variability, functional demands, and the management practices employed by local farmers.

Finally, Creole cattle from the Ayacucho region represent an important source of food and draught power, having adapted to local conditions for approximately five centuries. Consequently, these animals are highly resilient to abrupt climatic changes, capable of utilizing low-quality forage, able to move efficiently on steep mountainous terrain, and capable of producing meat and milk efficiently when provided with adequate nutrition, health management, and welfare conditions.

## 5. Conclusions

The Creole cattle of the department of Ayacucho exhibit high morphological harmony, as evidenced by the positive and significant correlations among the majority of zoometric variables, and moderate uniformity, reflected in the coefficients of variation recorded across body measurements. The morphostructural variability, as evidenced by significant differences in body measurements and zoometric indices among the three identified biotypes, confirms their value as a zoogenetic resource adapted to the diversity of agroecosystems of the Peruvian Andes. Three well-differentiated biotypes were identified, with a predominance of medium- to small-sized animals, demonstrating the presence of subpopulations with distinct conformation and likely different productive aptitudes. Biotype 1 comprises heavier, more compact and robust animals, suggesting a greater beef production aptitude, followed by Biotype 2, which shows a stronger dual-purpose tendency, whereas Biotype 3 includes lighter and more slender animals with relatively strong limbs, indicating greater efficiency for locomotion in rugged terrain. The variability of phaneroptic traits may be associated with low directed selection pressure and a long adaptation period; however, these traits were not associated with the identified biotypes, suggesting that morphostructural differentiation is independent of qualitative traits in this Creole cattle population. Overall, these results highlight the high potential of high-Andean Creole cattle from Ayacucho for conservation programs and the rational use of their diversity in the development of local genotypes, owing to their morphostructural characteristics adapted to high-altitude conditions, low-quality pastures, cold climates, and rugged landscapes.

## Figures and Tables

**Table 1:** Phaneroptic characteristics recorded in Creole cattle.

Phaneroptic Traits	Evaluation Indicators	Reference
Coat color (CC)	Wide variability	[24]
Coat pattern/extension (CE)	Solid, bicolor, or tricolor	
Fronto-nasal profile (FNP)	Concave, convex, or straight	[9]
Horn type (HT)	Polled, twisted, long-horned, open-horned, short-horned, forward-pointing, or hooked-shaped	
Mucosal pigmentation (MP)	Light or dark	
Dorsolumbar line (DL)	Straight, concave, or convex	
Leg conformation (LC)	Normal or bow-legged	
Hoof pigmentation (HPg)	Light, light/dark or dark	
Teat type (TT)	Cylindrical, bottle-shaped, or funnel-shaped	[25]
Teat orientation (TD)	Parallel or divergent	
Teat color (TC)	Light, light/dark or dark	
Udder type (UT)	Pendulous or rounded	Rated by an experienced veterinarian
Udder pigmentation (UP)	Light, light/dark or dark	

**Table 2:** Zoometric measurements and their descriptions based on anatomical landmarks in Creole cattle.

Body Measurements	Description Based on Anatomical Landmarks	Reference
Live weight (LW)	Weight estimated using a bovine weight tape	[26]
Horn length (HL), cm	From the proximal base of the horn to the distal tip	[13,27,28]
Neck length (NL), cm	From the right scapulohumeral joint to the left scapulohumeral joint	[13]
Neck circumference (NC), cm	Circumference measured passing through the seventh cervical vertebra	[26]
Withers height (WH), cm	From the ground to the withers	[27,28]
Withers width (WW), cm	Distance between the medial borders of the left and right scapula	[27]
Chest width (CW), cm	Distance between the right and left scapulohumeral joints	[28]
Thoracic perimeter (TP), cm	Circumference passing through the sternum and the seventh thoracic vertebra	[27,28]
Body length (BL), cm	From the scapulohumeral joint to the pin bone	[27,28]
Body depth (BD), cm	Vertical depth measured at the thoracic region	[29]
Abdominal perimeter (AP), cm	Circumference passing through the first lumbar vertebra and the 13th rib	[13]
Hip height (HH), cm	From the ground to the midpoint between both iliac tuberosities	[27]
Rump width (RW), cm	Distance between the two iliac tuberosities	[27,28]
Rump length (RL), cm	From the iliac tuberosity to the ischial tuberosity	[28]
Hip width (HW), cm	Distance between the right and left coxofemoral joints	[26]
Udder depth (UD), cm	From the proximal to the distal portion of the median suspensory ligament	[25,29]
Teat length (TL), cm	From the proximal to the distal end of the teat	[25]
Teat diameter (TD), cm	Distance between the distal lateral borders of the teat	[25]
Fore cannon perimeter (FCP), cm	Circumference of the mid-metacarpus	[27]
Hind cannon perimeter (HCP), cm	Circumference of the mid-metatarsus	[26,27]

**Table 3:** Calculation of zoometric indices in Creole cattle.

Zoometric Indices	Equation
Ethnological indices	
Body Index (BIx)	Body length/thoracic perimeter × 100
Pelvic Index (PIx)	Rump width/rump length × 100
Proportionality Index (PrIx)	Withers height/body length × 100
Productive indices	
Compactness Index (ComIx)	Live weight/withers height × 100
Dactylo-Thoracic Index (DTIx)	Fore cannon perimeter/thoracic perimeter × 100
Functional indices	
Transversal Pelvic Index (TPIx)	Rump width/withers height × 100
Longitudinal Pelvic Index (LPIx)	Rump length/withers height × 100
Relative Cannon Thickness Index (RCTIx)	Fore cannon perimeter/withers height × 100
Cannon Load Index (CLIx)	Fore cannon perimeter/live weight × 100

**Table 4:** Mean ± SE of zoometric variables of Creole adult cows from the districts of Chuschi and Chipao, Ayacucho Department.

Variables	Chuschi	Chipao	*p*-Value	Total	CV (%)
Horn Length (cm)	26.15 ± 0.84	25.54 ± 0.72	0.591	25.87 ± 0.56	26.32
Neck Length (cm) *	37.94 ± 0.55 a	33.93 ± 0.38 b	<0.001	36.05 ± 0.38	13.00
Neck Circumference (cm)	83.86 ± 0.79 a	79.41 ± 0.87 b	<0.001	81.75 ± 0.61	9.26
Withers Height (cm)	117.69 ± 0.68 b	119.97 ± 0.71 a	0.022	118.77 ± 0.50	5.20
Withers Width (cm) *	31.00 ± 0.52 a	29.36 ± 0.4 b	0.017	30.22 ± 0.34	13.92
Chest Width (cm) *	35.77 ± 0.47	35.92 ± 0.35	0.935	35.84 ± 0.30	10.36
Thoracic Perimeter (cm)	165.62 ± 1.18 a	162.29 ± 1.09 b	0.041	164.04 ± 0.82	6.18
Body Length (cm)	132.98 ± 0.94	134.92 ± 1.13	0.186	133.90 ± 0.73	6.78
Body Depth (cm) *	63.10 ± 0.56	64.90 ± 0.55	0.281	64.48 ± 0.39	7.56
Abdominal Perimeter (cm)	193.72 ± 1.76	193.27 ± 2.43	0.882	193.51 ± 1.48	9.46
Rump Height (cm)	123.93 ± 0.70	125.58 ± 0.69	0.096	124.71 ± 0.50	4.93
Rump Width (cm) *	39.81 ± 0.42	39.12 ± 0.29	0.095	39.49 ± 0.26	8.19
Rump Length (cm) *	44.30 ± 0.42	45.23 ± 0.33	0.116	44.74 ± 0.27	7.55
Hip Width (cm) *	43.89 ± 0.43	43.40 ± 0.35	0.425	43.66 ± 0.28	8.04
Udder Depth (cm)	29.51 ± 0.74	29.33 ± 0.59	0.848	29.41 ± 0.46	18.23
Teat Length (cm) *	5.51 ± 0.18 a	4.80 ± 0.15 b	0.004	5.13 ± 0.12	26.94
Teat Diameter (cm) *	2.17 ± 0.06	2.16 ± 0.06	0.854	2.17 ± 0.04	22.57
FCBC (cm) *	16.52 ± 0.11 a	15.68 ± 0.13 b	<0.001	16.13 ± 0.09	7.11
RCBC (cm) *	18.57 ± 0.13 a	17.69 ± 0.14 b	<0.001	18.16 ± 0.10	7.01
Thigh Width (cm) *	11.89 ± 0.44	11.07 ± 0.20	0.284	11.39 ± 0.21	20.32
Live weight (kg) *	410.09 ± 8.09 a	320.25 ± 6.60 b	<0.001	368.76 ± 6.45	21.41

(*) Variables analyzed using the nonparametric Mann–Whitney U tests. Different letters (a,b) within rows indicate significant differences (*p* < 0.05) according to the *t*-test. FCBC: Front Cannon Bone Circumference; RCBC: Rear Cannon Bone Circumference; CV: coefficient of variation.

**Table 5:** Rotated component matrix of the first five dimensions of explained variance of zoometric variables in Creole cows.

Variable	Dimension				
	1	2	3	4	5
Neck Circumference	−0.179	−0.437	−0.245	−0.394	−0.242
Withers Height	−0.275	0.156	0.509	−0.141	−0.128
Withers Width	−0.213	−0.355	0.186	0.075	0.520
Chest Width	−0.251	0.175	−0.266	0.019	0.417
Thoracic Perimeter	−0.291	−0.133	−0.035	−0.046	0.016
Body Length	−0.305	0.062	−0.027	0.051	−0.254
Body Depth	−0.280	0.001	0.089	0.149	0.295
Abdominal Perimeter	−0.293	0.050	−0.104	−0.197	0.391
Rump Height	−0.270	0.234	0.451	−0.141	−0.176
Rump Width	−0.222	−0.017	−0.09	0.688	−0.142
Rump Length	−0.243	0.287	0.182	0.051	−0.056
Hip Width	−0.236	0.222	−0.288	−0.463	−0.055
Udder Depth	−0.171	0.461	−0.463	0.151	−0.120
FCBC	−0.285	−0.342	−0.045	0.097	−0.232
RCBC	−0.308	−0.296	−0.102	0.101	−0.225

FCBC: Front Cannon Bone Circumference, RCBC: Rear Cannon Bone Circumference.

**Table 6:** PERMANOVA analysis of clusters or biotypes formed by Creole cows according to their zoometric variables.

Source of Variation	Degrees of Freedom	Sum of Squares	R^2^	F	*p*-Value
Cluster	2	51.261	0.43489	58.103	0.001 ***
Residual	151	66.609	0.56511		
Total	153	117.870	1.00000		

(***) Indicates highly significant differences among clusters at *p* < 0.001.

**Table 7:** Zoometric variables (mean ± SE) of biotypes of Creole cows from the high Andean region of Ayacucho Department.

Variables	Biotypes			*p*-Value
	1	2	3	
N	26	74	54	
Horn Length (cm)	24.00 ± 1.14	26.46 ± 0.78	25.93 ± 1.04	0.311
Neck Length (cm) *	37.00 ± 0.91	36.14 ± 0.54	35.47 ± 0.65	0.648
Neck Circumference (cm)	85.77 ± 1.37 a	82.96 ± 0.82 a	78.17 ± 0.97 b	<0.001
Withers Height (cm)	126.38 ± 0.92 a	119.50 ± 0.50 b	114.11 ± 0.65 c	<0.001
Withers Width (cm) *	34.77 ± 1.07 a	30.15 ± 0.35 b	28.13 ± 0.43 c	<0.001
Chest Width (cm) *	40.00 ± 0.41 a	36.14 ± 0.37 b	33.43 ± 0.41 c	<0.001
Thoracic Perimeter (cm)	174.42 ± 1.87 a	166.38 ± 0.90 b	155.83 ± 0.89 c	<0.001
Body Length (cm)	145.04 ± 1.20 a	135.28 ± 0.79 b	126.63 ± 0.85 c	<0.001
Body Depth (cm) *	69.88 ± 0.54 a	65.58 ± 0.52 b	60.48 ± 0.33 c	<0.001
Abdominal Perimeter (cm)	215.08 ± 2.90 a	197.26 ± 1.44 b	177.98 ± 1.73 c	<0.001
Rump Height (cm)	132.62 ± 1.20 a	125.05 ± 0.49 b	120.43 ± 0.56 c	<0.001
Rump Width (cm) *	42.69 ± 0.59 a	39.72 ± 0.31 b	37.63 ± 0.38 c	<0.001
Rump Length (cm) *	47.92 ± 0.40 a	45.42 ± 0.27 b	42.28 ± 0.47 c	<0.001
Hip Width (cm) *	46.81 ± 0.81 a	44.11 ± 0.33 b	41.52 ± 0.35 c	<0.001
Udder Depth (cm)	33.00 ± 1.09 a	29.88 ± 0.66 b	27.11 ± 0.65 c	<0.001
Teat Length (cm) *	5.86 ± 0.31 a	5.01 ± 0.16 b	4.96 ± 0.20 b	0.033
Teat Diameter (cm) *	2.34 ± 0.10	2.13 ± 0.05	2.13 ± 0.09	0.138
FCBC (cm) *	17.50 ± 0.19 a	16.24 ± 0.09 b	15.31 ± 0.13 c	<0.001
RCBC (cm) *	19.73 ± 0.21 a	18.32 ± 0.10 b	17.18 ± 0.13 c	<0.001
Thigh Width (cm) *	12.61 ± 0.50 a	11.36 ± 0.32 b	10.71 ± 0.30 b	0.010
Live weight (kg) *	449.75 ± 15.44 a	378.94 ± 7.67 b	319.19 ± 8.34 c	<0.001

FCBC: Front Cannon Bone Circumference; RCBC: Rear Cannon Bone Circumference. Different letters (a,b,c) within rows indicate significant differences at *p* < 0.05 according to the Duncan test. (*) Variables analyzed using the nonparametric Kruskal–Wallis and Mann–Whitney U tests with Bonferroni correction.

**Table 8:** Zoometric indices of biotypes of Creole cows from the high Andean region of the Ayacucho Department.

Zoometric Indices	Biotypes			*p*-Value	Total
	1	2	3		
N	26	74	54		154
Ethnological indices					
Body index	83.41 ± 1.20	81.45 ± 0.60	81.37 ± 0.63	0.203	81.75 ± 0.42
Pelvic index *	89.28 ± 1.56	87.63 ± 0.80	89.86 ± 1.76	0.750	88.69 ± 0.77
Proportionality index *	87.25 ± 0.80 b	88.57 ± 0.68 ab	90.30 ± 0.72 a	0.020	88.95 ± 0.44
Productive indices					
Compactness index *	356.37 ± 12.57 a	318.04 ± 6.87 b	279.94 ± 7.30 c	<0.001	310.46 ± 5.12
Dactylo-thoracic index	10.06 ± 0.14	9.78 ± 0.07	9.83 ± 0.08	0.124	9.84 ± 0.05
Functional indices					
Transversal pelvic index *	33.84 ± 0.57	33.28 ± 0.29	33.04 ± 0.39	0.707	33.29 ± 0.22
Longitudinal pelvic index *	37.96 ± 0.37	38.05 ± 0.26	37.10 ± 0.44	0.233	37.70 ± 0.21
Relative cannon thickness index	13.87 ± 0.19	13.61 ± 0.10	13.44 ± 0.15	0.171	13.59 ± 0.08
Cannon load index	3.97 ± 0.11 c	4.41 ± 0.09 b	4.93 ± 0.11 a	<0.001	4.53 ± 0.07

Different letters (a,b,c) within rows indicate significant differences at *p* < 0.05 according to the Duncan test. (*) Variables analyzed using the nonparametric Kruskal–Wallis and Mann–Whitney U tests with Bonferroni correction.

**Table 9:** Association between categorical variables and identified biotypes of Creole cows from the high Andean region of Ayacucho Department.

Variable	Category	Biotypes			*p*-Value *	Total
		1	2	3		
District	Chuschi	13 (50.0)	40 (54.1)	28 (51.9)	0.930	81 (52.6)
	Chipao	13 (50.0)	34 (45.9)	26 (48.1)		73 (47.4)
Body score	2.25	0 (0)	2 (2.7)	6 (11.1)	0.208	8 (5.2)
	2.50	11 (42.3)	38 (51.4)	28 (51.9)		77 (50.0)
	2.75	5 (19.2)	13 (17.6)	6 (11.1)		24 (15.6)
	3.00	9 (34.6)	17 (23.0)	9 (16.7)		35 (22.7)
	3.50	1 (3.8)	4 (5.4)	5 (9.3)		10 (6.5)

(*) Independence of variables analyzed using the χ^2^ test (*p* < 0.05).

**Table 10:** Frequency of phaneroptic variables of biotypes of Creole cows from the high Andean region of Ayacucho Department.

Variable	Category	Biotypes			*p*-Value *	Total
		1	2	3		
Coat pattern	Solid	7 (26.9)	20 (27.0)	20 (37.0)		47 (30.5)
	Bicolor	17 (65.4)	51 (68.9)	28 (51.9)	0.303	96 (62.3)
	Tricolor	2 (7.7)	3 (4.1)	6 (11.1)		11 (7.1)
Fronto-nasal profile	Concave	0 (0)	4 (5.4)	5 (9.3)	0.249	9 (5.8)
	Straight	26 (100)	70 (94.6)	49 (90.7)		145 (94.2)
Horn type	Polled	2 (7.7)	4 (5.4)	1 (1.9)	0.471	7 (4.5)
	Twisted	1 (3.8)	5 (6.8)	2 (3.7)		8 (5.2)
	Long-horned	3 (11.5)	23 (31.1)	14 (25.9)		40 (26.0)
	Open-horned	5 (19.2)	10 (13.5)	13 (24.1)		28 (18.2)
	Short-horned	14 (53.8)	24 (32.4)	21 (38.9)		59 (38.3)
	Forward-pointing	0 (0)	1 (1.4)	1 (1.9)		2 (1.3)
	Hook-shaped	1 (3.8)	7 (9.5)	2 (3.7)		10 (6.5)
Dorsolumbar line	Concave	3 (11.5)	8 (10.8)	8 (14.8)	0.786	19 (12.3)
	Straight	23 (88.5)	66 (89.2)	46 (85.2)		135 (87.7)
Mucosal pigmentation	Light	1 (3.8)	6 (8.1)	3 (5.6)	0.706	10 (6.5)
	Dark	25 (96.2)	68 (91.9)	51 (94.4)		144 (93.5)
Udder type	Pendulous	6 (23.1)	17 (23.0)	9 (16.7)	0.652	32 (20.8)
	Rounded	20 (76.9)	57 (77.0)	45 (83.3)		122 (79.2)
Udder pigmentation	Light	18 (69.2)	47 (63.5)	32 (59.3)	0.579	97 (63.0)
	Light/Dark	3 (11.5)	16 (21.6)	9 (16.7)		28 (18.2)
	Dark	5 (19.2)	11 (14.9)	13 (24.1)		29 (18.8)
Teat type	Bottle-shaped	7 (26.9)	9 (12.2)	14 (25.9)	0.029	30 (19.5)
	Cylindrical	14 (53.8)	43 (58.1)	18 (33.3)		75 (48.7)
	Funnel-shaped	5 (19.2)	22 (29.7)	22 (40.7)		49 (31.8)
Teat orientation	Divergent	9 (34.6)	23 (31.1)	18 (33.3)	0.933	50 (32.5)
	Parallel	17 (65.4)	51 (68.9)	36 (66.7)		104 (67.5)
Teat color	Light/Dark	7 (26.9)	11 (14.9)	9 (16.7)	0.103	27 (17.5)
	Light	16 (61.5)	39 (52.7)	23 (42.6)		78 (50.6)
	Dark	3 (11.5)	24 (32.4)	22 (40.7)		49 (31.8)
Leg conformation	Normal	24 (92.3)	61 (82.4)	44 (81.5)	0.428	129 (83.8)
	Bow-legged	2 (7.7)	13 (17.6)	10 (18.5)		25 (16.2)
Hoof pigmentation	Light	1 (3.8)	1 (1.4)	4 (7.4)	0.413	6 (3.9)
	Light/Dark	2 (7.7)	4 (5.4)	5 (9.3)		11 (7.1)
	Dark	23 (88.5)	69 (93.2)	45 (83.3)		137 (89.0)

(*) Independence of variables analyzed using the χ^2^ test (*p* < 0.05).

**Table 11:** Frequency of coat colors of biotypes of Creole cows from the high Andean region of Ayacucho Department.

Coat Color	Biotypes			Total
	1	2	3	
N	26	74	54	
White	1 (3.85)	1 (1.35)	2 (3.70)	4 (2.60)
Callejón	4 (15.38)	2 (2.70)	-	6 (3.90)
Red	3 (11.54)	8 (10.81)	7 (12.96)	18 (11,69)
Dark roan	-	4 (5.41)	9 (16.67)	13 (8.44)
Black	4 (15.38)	16 (21.62)	7 (12.96)	27 (17.53)
Jet black	1 (3.85)	-	6 (11.11)	7 (4.55)
Dull black	3 (11.54)	4 (5.41)	4 (7.41)	11 (7.14)
Other *	1 (3.85)	8 (10.81)	4 (7.41)	13 (8.44)
Qosca	6 (23.08)	18 (24.32)	7 (12.96)	31 (20.13)
Qosne	-	6 (8.11)	3 (5.56)	9 (5.84)
Roman	2 (7.69)	2 (2.70)	-	4 (2.60)
Roan	1 (3.85)	5 (6.76)	5 (9.26)	11 (7.14)

(*) The category “Other” includes less frequent coat colors such as Bayo, Pillca, Puca moro, Puca pillca, Qallawa, Tres Pelos, Ccarhua, and Umara.

## Data Availability

The dataset is available from the corresponding author upon reasonable request.
